# DDR1 promotes metastasis of cervical cancer and downstream phosphorylation signal via binding GRB2

**DOI:** 10.1038/s41419-024-07212-5

**Published:** 2024-11-20

**Authors:** Jin Zhang, Aynuer Maimaiti, Xihan Chang, Pengcheng Sun, Xiaohan Chang

**Affiliations:** 1 Department of Obstetrics and Gynecology, Shengjing Hospital of China Medical University, Shenyang, Liaoning China; 2 Department of Obstetrics and Gynecology, Tacheng Hospital of China Medical University, Tacheng, Xinjiang Uygur Autonomous Region China; 3 The Second Clinical College of China Medical University, Shenyang, Liaoning China

**Keywords:** Cancer genomics, Cervical cancer

## Abstract

Cervical cancer is a leading cause of cancer-related death among women and its recurrence and metastasis poses challenges to treatment. Discoidin domain receptor 1 (DDR1) was associated with cellular migration and invasion in several types of cancers. However, its function in cervical cancer is still unclear. In this study, we found that DDR1 was significantly more expressed in cervical cancer samples than in normal tissues. SRY-Box transcription factor 2 (SOX2), a known oncogene in cervical cancer, showed a positive correlation with DDR1 and regulated DDR1 transcription, contributing to the elevated expression of DDR1 in cervical cancer. Regarding the function of DDR1 in cervical cancer, the overexpression of DDR1 caused an increase in the migration, invasion, and epithelial-mesenchymal transition (EMT) of cervical cancer cells. In contrast, cervical cancer cells with reduced DDR1 expression exhibited a lower migration rate, fewer invasive cells, and decreased levels of EMT markers. In vivo, mice injected with cervical cancer cells with overexpressed DDR1 showed more pulmonary metastasis and nodule number. Opposite results were found in mice injected with DDR1 silenced cervical cancer cells. Since DDR1 can cause phosphorylation of downstream targets, a phosphorylation omics was employed to reveal the downstream targets of DDR1, including eukaryotic translation initiation factor 4E binding protein 1 and EPH receptor A2. Furthermore, DDR1 bound directly with Src homology 2 domain of growth factor receptor bound protein 2 (GRB2) which mediated the function of DDR1 in the malignant behaviors of cervical cancer and the phosphorylation of downstream targets. In conclusion, DDR1 binds directly to GRB2 and then affects downstream phosphorylation signals, ultimately exacerbating the metastasis of cervical cancer cells. This work sheds light on the mechanism by which DDR1 functions in cervical cancer cells, providing therapeutic strategy for the treatment of cervical cancer.

## Introduction

Cervical cancer is mainly caused by high-risk infection of the human papilloma virus [1], and has become the most commonly malignant tumor of the female reproductive system [2], with approximately 342,000 deaths each year [3]. Recently, due to the increased use of early tests, such as CryoPen and thermal coagulation technologies, the incidence and mortality have declined [4]. However, the overall prognosis of patients, who have advanced or recurrent metastatic cervical cancer, remains poor [5]. Therefore, it is necessary to explore the molecular mechanisms of cervical cancer development.

Discoidin domain receptor 1 (DDR1) is a member of receptor tyrosine kinase family and widely expressed in epithelial cells [6]. Current studies indicated that DDR1 participates in key cellular processes, including survival, proliferation, migration and invasion [7]. Besides, increased DDR1 is known to be associated with the progression and poor prognosis of non-small cell lung carcinoma, hepatocellular carcinoma, bladder cancer, and oral squamous cell carcinoma, indicating an important role of DDR1 in in tumor progression [8–11]. Nevertheless, the function and detailed mechanism of DDR1 in the development of cervical cancer remain obscure.

SRY-Box transcription factor 2 (SOX2) is a transcription factor and involved in the regulation of embryonic development and the determination of stem cell fate [12]. As a marker of cancer stem cells, SOX2 is related to a worse prognosis in advanced cancer, and is considered as a potential oncogenic factor in cervical cancer [13]. In the other hand, as a transcription factor, SOX2 regulated gene transcription and then changed gene expression [14]. Interestingly, based on website prediction (https://jaspar.genereg.net/analysis) and previous study (https://www.ncbi.nlm.nih.gov/geo/), there were binding sites of SOX2 in the promoter region of DDR1, and when SOX2 was absent, a reduced expression of DDR1 was observed. Therefore, we speculated that SOX2 could bind to the promoter region of DDR1, thereby influencing the transcription of DDR1.

Receptor tyrosine kinases play critical roles in an assortment of cellular processes, while dysregulation of receptor tyrosine kinase signaling causes a variety of human diseases, most notably, cancers [15]. Growth factor receptor bound protein 2 (GRB2), as an adaptor protein which links the receptor tyrosine kinases and the downstream targets, is required by almost all receptor tyrosine kinases for signal transduction and plays a positive role in transduction [16], such as activating Protein Kinase B and mitogen-activated protein kinase signaling pathways [17, 18]. However, whether GRB2 serves as an adaptor protein mediating the role of DDR1 in cervical cancer remains unknown.

Here, we sought to emphatically explore the function of DDR1 in cervical cancer. Mechanistically, whether SOX2 had a regulatory effect on DDR1 transcription and whether GRB2 mediated DDR1 function in cervical cancer were explored.

## Materials and methods

### Clinical samples

All procedures involved in human subjects were complied with Declaration of Helsinki and approved by the Ethics Committee of Shengjing Hospital of China Medical University. Each patient signed informed consent prior to participating in the study. The sample size was estimated based on G*Power (3.1.9.7).

### Cell treatment

Cervical cancer cell lines, including C33A and SiHa, and HEK293T cells were purchased from the iCell Bioscience Inc. (Shanghai, China) that passed the STR and mycoplasma contamination test. The detailed information of cell culture was listed at Supplementary Information (SI).

Cervical cancer cells were infected with the lentivirus (inducible expression system), including DDR1 short hairpin RNA (shDDR1) and DDR1 overexpression (oeDDR1), and then puromycin (Macklin, Shanghai, China) was used to screen stably infected cell lines. These vectors were purchased from Hunan Fenghui Biotechnology Co., Ltd. (Changsha, China). After screening, doxycycline (DOX, 1 μg/ml, Macklin) was added to induce target gene expression.

Small interfering RNA targeting SOX2 (siSOX2) or GRB2 (siGRB2), and SOX2 overexpression (oeSOX2) plasmid were used to transfected cells, followed with DOX treatment. In addition, HEK293T cells were transfected with flag-GRB2 and myc-DDR1 or myc-DDR1-K618A, which is an inactivated mutant of DDR1. Next, myc-DDR1 and flag-GRB2 or flag-GRB2 with missing domains, including GRB2ΔnSH3 (N-terminal Src homology 3 domain deficiency), GRB2ΔSH2 (Src homology 2 domain deficiency), GRB2ΔcSH3 (C-terminal Src homology 3 domain deficiency), were co-transfected into HEK293T cells.

### Wound healing assay and transwell assay

Before the assay, the medium was replaced with serum-free medium and treated with mitomycin C (Sigma, St. Louis, MO, USA) for 60 min. The “wound” was created by a 200 μL pipette tip. The wound images were captured using an IX53 microscope (OLYMPUS, Tokyo, Japan).

For transwell assay, 300 μL of cell suspension (serum-free medium) was seeded in the upper chamber, and the lower chamber was added with 700 μL medium (containing 10% FBS). After cultivation for 24 h, 4% polyformaldehyde (Aladdin, Shanghai, China) was used for fixation and 0.4% crystal violet (Amresco, Solon, OH, USA) was used for staining, subsequently. The cells were observed under microscope.

### Luciferase reporter assay

The differently truncated promoters of DDR1 were constructed into PGL3-basic vector and then was co-transfected with oeSOX2 and pRL-TK plasmids into HEK293T cells. The activity of luciferase was determined with the Luciferase Assay Kit (KeyGEN, Nanjing, China) according to the manufacturer’s instructions.

### Lung metastasis

All animal experiments were conducted in compliance with the Guideline for the Care and Use of Laboratory Animals and Basel Declaration, and were approved by the Ethics Committee of Shengjing Hospital of China Medical University. The detailed environmental conditions were written in SI. The sample size was estimated based on previous experience in our laboratory and previous studies by others [19, 20].

After one week of adaptive feeding, mice were assigned to different groups by block randomization, where equal numbers of every group were presented. SiHa cells with stable expression of shDDR1 or oeDDR1 were injected into the tail vein of female BALB/c nude mice (six-week-old), and then DOX (200 μg/ml) was immediately provided in drinking water. Six weeks after the injection, small animal imaging system was used to observe the cell metastasis and then the mice were sacrificed. The lungs were harvested for the subsequent experiments. Blinding was performed and the tester was blind to experimental groups.

### Hematoxylin-eosin (HE) staining

The tissues were collected and made into 5-μm-thick sections. After being dewaxed and hydrated, the sections were stained with hematoxylin (Solarbio, Beijing, China) for 5 min and then soaked in eosin (Sangon, Shanghai, China) for 3 min. BX53 microscope (OLYMPUS) was used for the observation and photography.

### Immunohistochemistry

After antigen retrieval, the sections were blocked with bovine albumin (Sangon). Then the sections were incubated with antibodies (details were shown in SI). Target proteins were visualized using diaminobenzidine (DAB, MXB Biotechnologies, Fuzhou, China), followed by counterstaining with hematoxylin (Solarbio). The dyeing effect was observed under BX53.

### Immunofluorescent staining

Cell slides were made and then they were fixed in 4% paraformaldehyde for 15 min, permeabilized in 0.1% tritonX-100 (Beyotime, Shanghai, China) for 30 min, and blocked in 1% BSA (Sangon) for 15 min. Cell slides were then incubated with corresponding antibodies (detailed information was provided in SI). Finally, cell nucleus was stained by 4’,6-diamidino-2-phenylindole (Aladdin), and immunofluorescence images were obtained by BX53.

### Real-time polymerase chain reaction (RT-PCR)

Total RNA was isolated by using TRIpure (Bioteke, Beijing, China). BeyoRT™ II M-MLV reverse transcriptase (RNase H-) (Beyotime, Shanghai, China) was utilized to generate cDNAs through reverse transcription and SYBR Green and 2×Taq PCR MasterMix (Solarbio) were used for quantitative PCR through Exicycler^TM^ 96 fluorescence quantitative instrument (Bioneer, Daejeon, Korea). The primer sequences were showed in SI. Relative mRNA levels were normalized to GAPDH and were calculated by the 2^−ΔΔCt^ method.

### Chromatin immunoprecipitation (ChIP) assay

A ChIP assay kit (Beyotime) was used according to the manufacturer’s protocol. For ChIP, 1 μg SOX2 antibody (Cell Signaling Technology, Cat. No.23064) and IgG antibody (Beyotime, Cat. No.A7001) which was considered as a negative control, were used. The immunoprecipitated DNA was verified by PCR following with electrophoresis.

### DNA pull-down

A DNA pulldown kit (BersinBio, Guangdong, China) was used according to the manufacturer’s instructions (details see SI).

### Western blot analysis

Total protein was extracted and then quantified with a bicinchoninic acid assay kit (Solarbio). The protein sample was loaded on the SDS-PAGE for electrophoresis, and transferred onto PVDF membranes. After being blocked for 60 min, the membranes were incubated with primary antibodies (details in SI) at 4 °C overnight. Subsequently, the membranes were incubated with secondary antibodies at 37 °C for 60 min. The ECL solution was used to visualize the blots.

### co-IP assay

Total protein was extracted and quantified. Pierce® Co-Immunoprecipitation Kit (Thermo Scientific) was used according to the manufacturer’s instructions following with western blot analysis. Detailed antibodies were written in SI.

### Bioanalysis

The data provided by Gene Expression Profiling Interactive Analysis (GEPIA, http://gepia.cancer-pku.cn/index.html) and GSE127265, which was downloaded from the Gene Expression Omnibus database (GEO, http://www.ncbi.nlm.nih.gov/geo) was used to analyze the differentially expressed genes (DEGs) in cervical cancer. DEGs were defined as *P* < 0.05 and │log2FC│ > 1. The correlation analysis between different genes also was conducted by GEPIA.

The analysis of phosphoproteomics and qualitative proteomics were performed by Beijing Qinglian Baiao Biotechnology Co., Ltd (Beijing Qinglian Baiao Biotechnology Co., Ltd., Beijing, China). *P* < 0.05 and │log2FC│ > 1 represented the differentially phosphorylated protein (DPP). log2FC > 1 indicated the hyperphosphorylated protein, while log2FC < −1 indicated hypophosphorylated protein.

### Statistical analysis

GraphPad Prism was used for statistical analysis. Results were presented as the mean ± standard deviation. *P* < 0.05 was considered statistically significant. All data accepted Normality and Lognormality Tests and variance comparison, and are analyzed by Wilcoxon test, t test or one-way ANOVA.

## Results

### DDR1 was upregulated in cervical cancer

**Fig. 1:**
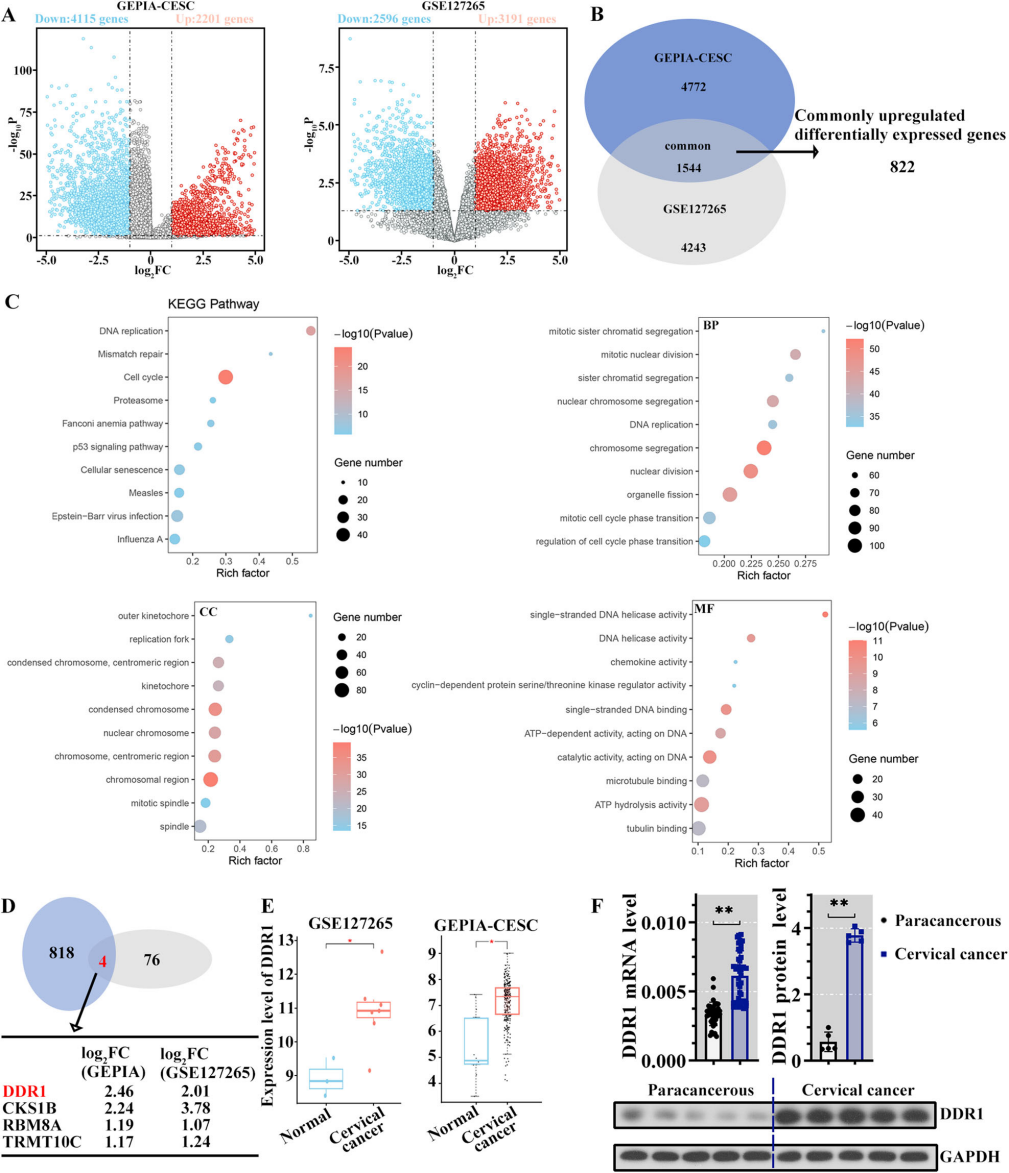
DDR1 was upregulated in cervical cancer. **A** Volcano plot of gene expression in normal and cervical cancer group. DEGs were defined as *P* < 0.05 and │log2FC│ > 1. **B** Results of Veen analysis of the differentially expressed genes between the two datasets. **C** Results of GO and KEGG enrichment analysis of common upregulated differentially expressed genes between the two datasets. **D** Venn diagram between commonly upregulated DEGs and SOX2 targets obtained from Cistrome Data Browser. **E** The expression of DDR1 was increased in cervical cancer samples. *P < 0.05 was considered statistically significant. **F** RT-PCR and western blot were used to detect the expression of DDR1 in clinical samples (*N* = 40; **P < 0.01). DDR1 discoidin domain receptor 1, GO gene ontology, KEGG Kyoto encyclopedia of genes and genome, DEGs differentially expressed gene, RT-PCR real-time polymerase chain reaction.

To determine the mRNA expression profiling more fully and accurately in the progression of cervical cancer, we used data from two databases (Fig. 1A). After taking the intersection, 1544 common DEGs were observed that includes 822 upregulated DEGs (Fig. 1B). Next, the upregulated common DEGs were focused, and they were enriched in cellular processes-related items (Fig. 1C). Our previous study [21] demonstrated that SOX2 promoted the progress of cervical cancer. In consideration of the regulating transcription function of SOX2, it was worth investigating what was regulated by it to promote cervical cancer development. Cistrome Data Browser (http://cistrome.org/db/#/) provided genes who were regulated by SOX2. GEPIA and GSE127265 showed the DEGs in cervical cancer. Venn diagram displayed the genes that were modulated by SOX2 and differently expressed in cervical cancer (Fig. 1D). Notably, there were only four genes and DDR1 attracted our attention the most (Fig. 1D). The increased DDR1 expression was also observed in cervical cancer clinical samples provided in this study. (Fig. 1F). These data suggested that DDR1 may be related to the development of cervical cancer.

### DDR1 was transcriptionally regulated by SOX2

**Fig. 2:**
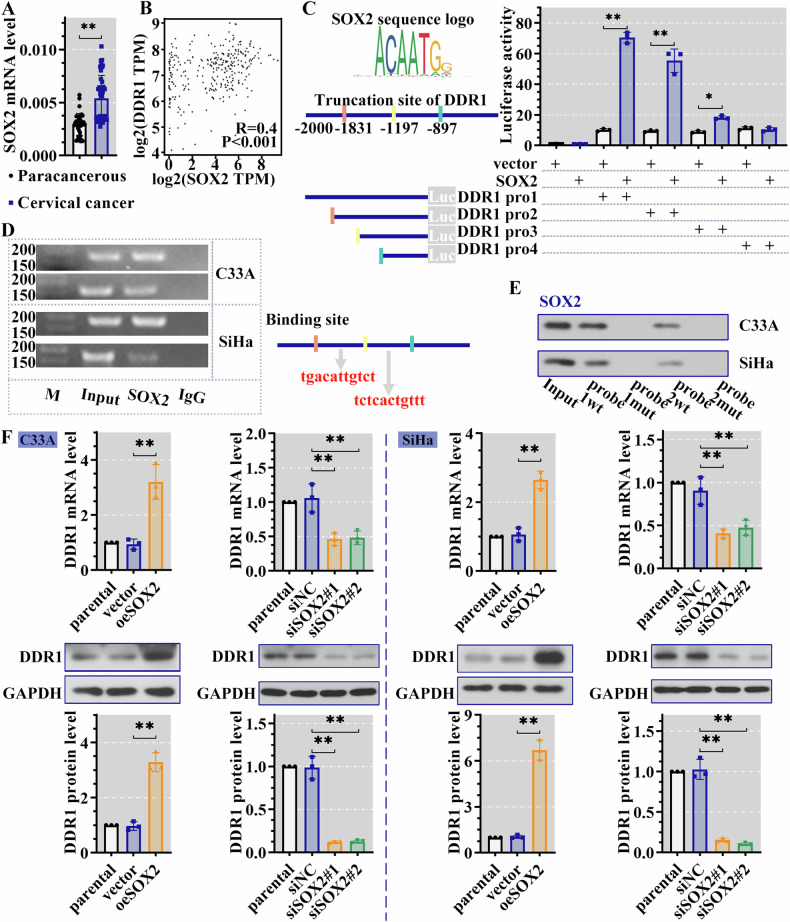
DDR1 was transcriptionally regulated by SOX2. **A** The SOX2 expression in clinical samples (*N* = 40) was measured by RT-PCR assay. **B** DDR1 was positively correlated with SOX2. **C** Luciferase reporter assay was used to verify the regulatory effect of SOX2 on DDR1 transcription. **D** The binding of SOX2 to DDR1 promoters was detected by ChIP. **E** DNA pull down was used to detect the binding of DDR1 promoters to SOX2. **F** The expression of DDR1 was measured by RT-PCR and western blot assay. Results were presented as the mean ± standard deviation (*N* = 3). *P < 0.05 was considered statistically significant; ** *P* < 0.01. DDR1 discoidin domain receptor 1, SOX2 SRY-Box transcription factor 2, RT-PCR real-time polymerase chain reaction, ChIP chromatin immunoprecipitation, DNA deoxyribonucleic acid.

In cervical cancer samples that we collected, the expression of DDR1 and SOX2 were increased, compared with paracancerous tissues (Figs. 1F and 2A). Information based on database and website prediction indicated that there seemed to be a connection between SOX2 and DDR1. In order to explore the relationship, the prediction in website was analyzed. Firstly. As shown in Fig. 2B, DDR1 was positively correlated with SOX2. Results in Jasper (https://jaspar.genereg.net/) showed that there may be binding sites of SOX2 in DDR1 promoter region, and luciferase assay was performed to clarify it. With the truncation of the promoter region, the activity gradually decreased in SOX2 overexpression cell. In the presence of DDR1 promoter region 4, there was no significant difference of luciferase activity between vector and SOX2 cell (Fig. 2C). It suggested that the most effective binding sites existed between promoter region 3 and 4. The subsequent results of ChIP and DNA pull down assay also verified that SOX2 combined the promoter of DDR1, thereby regulating the transcription of DDR1 (Fig. 2D, E). To investigate the effect of SOX2 on DDR1 transcription, we manipulated SOX2 level and tested the expression of DDR1. As shown in Supplementary Fig S1, the transfection of siSOX2 and oeSOX2 was successful. Overexpression of SOX2 increased the expression of DDR1, while SOX2 interference reduced the DDR1 expression (Fig. 2F). These results revealed that DDR1 transcription was promoted after SOX2 bound to DDR1 promoter.

### DDR1 promoted the metastasis of cervical cancer cell

**Fig. 3:**
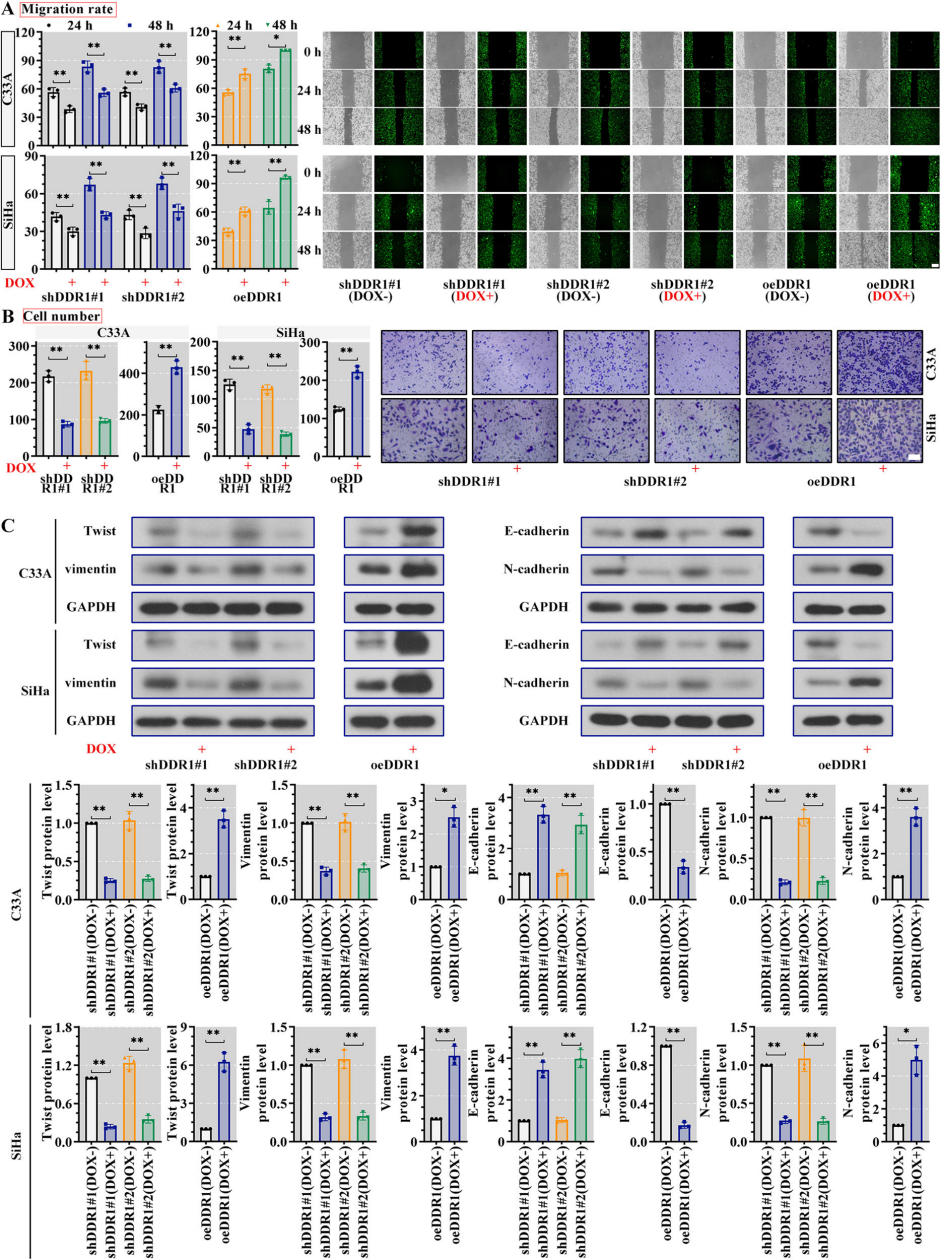
DDR1 promoted the metastasis of cervical cancer cell. **A** Cell migration was detected by wound healing assay (scale bar, 200 μm). **B** The invasion ability of cells was tested by transwell assay (scale bar, 100 μm). **C** Protein levels of Twist, vimentin, E-cadherin and N-cadherin were measured by western blot assay. Results were showed as the mean ± standard deviation (N = 3). *P < 0.05 was considered statistically significant; **P < 0.01. DDR1 discoidin domain receptor 1.

Next, we examined the effects of DDR1 on cervical cancer. Using the RT-PCR, we observed that lentivirus infection markedly changed the DDR1 mRNA level in the presence of DOX (Supplementary Fig. S2A). The protein results also suggested that DOX induced the DDR1 knockdown and overexpression after cell infection with corresponding lentivirus (Supplementary Fig. S2B). We found that as little as 4 h was sufficient to induce DDR1 expression. At the presence of DOX, reduced migration rate and invasive cell number were showed in shDDR1 cells, while increased migration rate and invasive cell number were showed in oeDDR1 cells (Fig. 3A, B). Twist, vimentin and N-cadherin were increased and E-cadherin was decreased in oeDDR1 cells and these changes were on the contrary in shDDR1 cells after DOX treatment (Fig. 3C). Meanwhile, the mesenchymal-like cells were increased and epithelial-like cells were reduced in oeDDR1 cells, while cells in DDR1 silencing group showed no signs of mesenchymal-like transformation, after adding DOX (Supplementary Fig. S3). Thus, overall results showed that DDR1 enhanced the migration and EMT of cervical cancer cell.

### DDR1 contributed to distant metastasis of cervical cancer in vivo

**Fig. 4:**
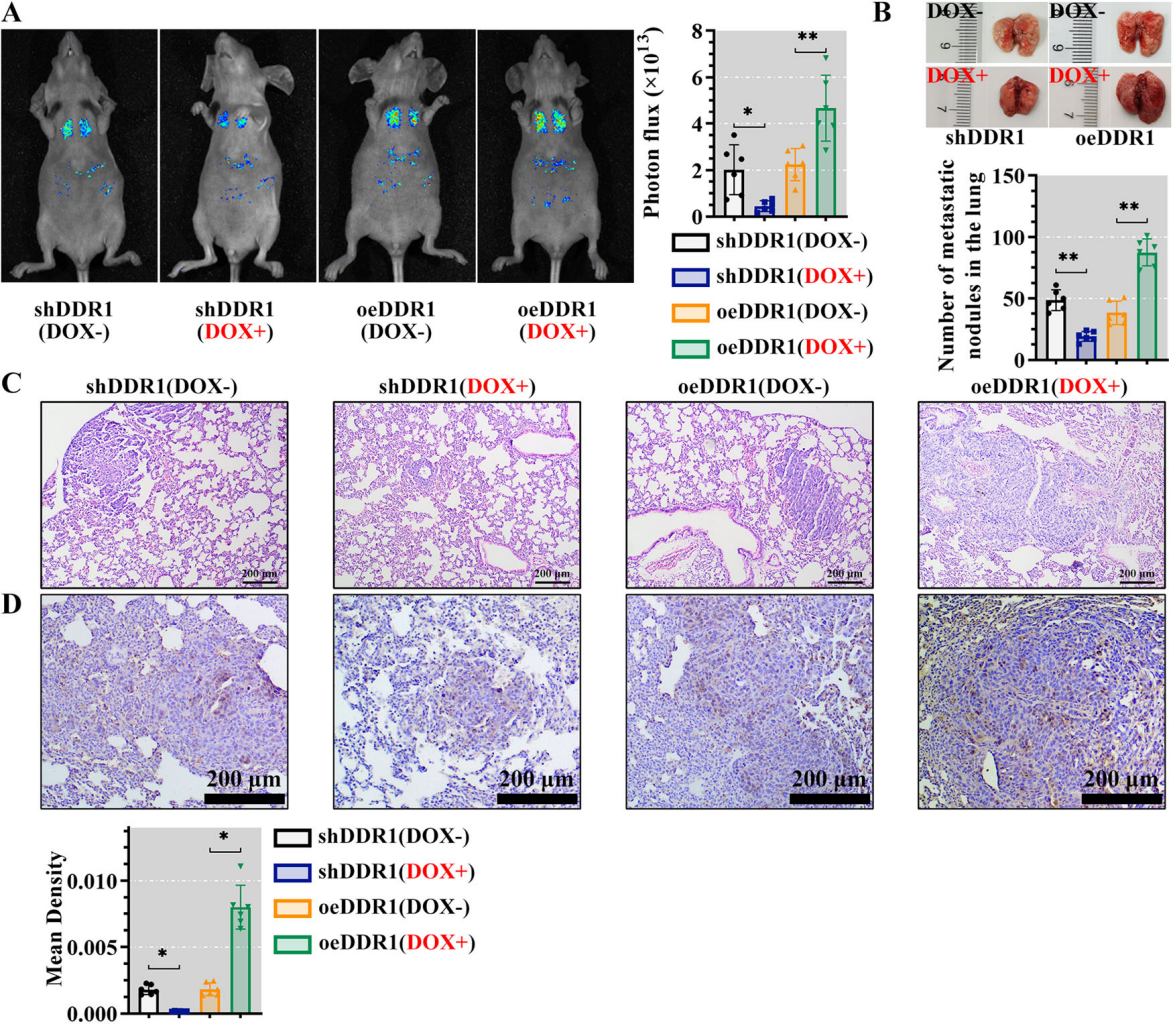
DDR1 contributed to distant metastasis in cervical cancer in vivo. **A** The small animal imaging system was used to observe the cell metastasis in vivo. **B** Representative lung images (left panel) and number of metastatic nodules in the lung (right panel). **C** HE staining was used to detect the lung histopathological changes (scale bar, 200 μm). **D** The expression of DDR1 in lung tissue was detected by IHC (scale bar, 200 μm). Data were appeared as the mean ± standard deviation (*N* = 6). *P < 0.05 was considered statistically significant; **P < 0.01. DDR1 discoidin domain receptor 1, HE hematoxylin-eosin, IHC.

Subsequently, we evaluated the in vivo metastatic potential of DDR1-expressing cervical cancer cells in the xenograft mouse model. After the tail vein injection, lower metastases were observed in shDDR1 mice (DOX+) and higher metastases were showed in oeDDR1 mice (DOX+), respectively. Remarkably, significant metastasis was observed in the lung (Fig. 4A). The number of metastatic nodules in lung sections from the oeDDR1 (DOX+) mice was more than that from the oeDDR1 (DOX-) group mice. Similarly, metastatic nodules were decreased in shDDR1 mice after DOX treatment, compared with DOX-untreated shDDR1 mice (Fig. 4B). Moreover, we observed greater nodules in DDR1 overexpressed mice; meanwhile, the DDR1 silenced mice had smaller nodules (Fig. 4C). Further, immunohistochemistry staining analysis showed higher DDR1 expression in lung tissues of mice with oeDDR1 cell injection treated by DOX than DOX-untreated oeDDR1 mice. Compared with shDDR1 mice in the absence of DOX, decreased DDR1 was expressed in shDDR1 mice with DOX (Fig. 4D). These results confirmed that DDR1 obviously promoted the distant metastasis of cervical cancer cells in vivo.

### Effect of DDR1 on downstream phosphorylation signal

**Fig. 5:**
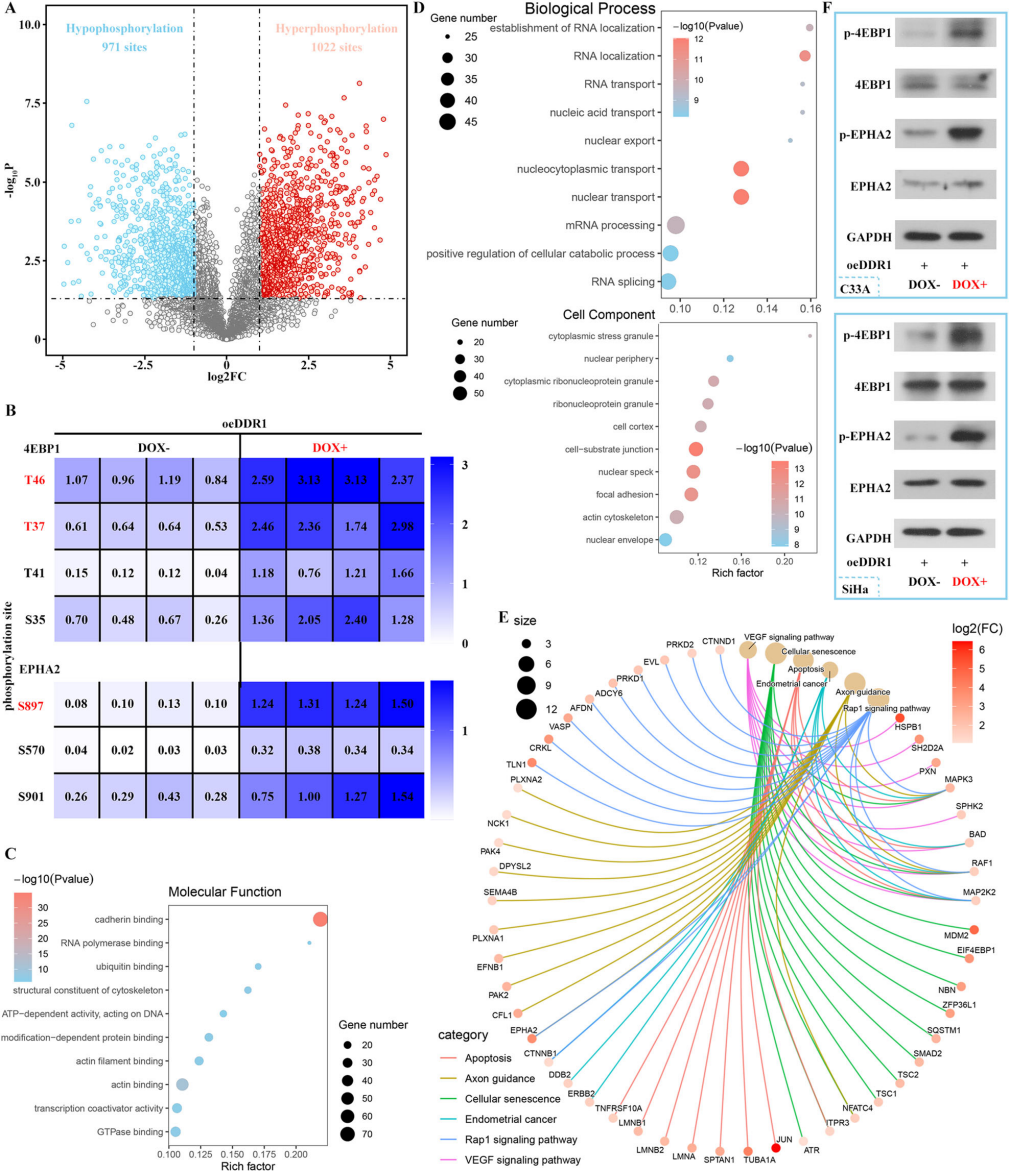
Effect of DDR1 on downstream phosphorylation signal. **A** Volcano plot of protein phosphorylation in DOX-treated and DOX-untreated oeDDR1 cells. **B** Heat map displayed phosphorylation of different sites in 4EBP1 and EPHA2. **C**–**E** Results of GO and KEGG enrichment analysis of the genes corresponding to hyperphosphorylated proteins. **F** Western blot was used to detect the level of p-4EBP1, 4EBP1, p-EPHA2 and EPHA2. Data were presented as the mean ± standard deviation. log2FC > 1 was defined as hyperphosphorylated protein, while log2FC < −1 was defined as hypophosphorylated protein, when *P* < 0.05. DDR1 discoidin domain receptor 1, DOX doxycycline, GO gene ontology, KEGG Kyoto encyclopedia of genes and genome, p-4EBP1 phosphorylation-eukaryotic translation initiation factor 4E binding protein 1, p-EPHA2 phosphorylation-EPH receptor A2.

To the best of our knowledge, receptor tyrosine kinase family can induce the phosphorylation of target protein. Therefore, we investigated the phosphorylation which was induced by DDR1 in the present study. After four hours of DDR1 induced-expression, the cells were collected for phosphorylated proteomics detection. As showed in Fig. 5A, 971 sites of hypophosphorylation and 1022 sites of hyperphosphorylation were observed and pathways enriched by them were shown in Fig. 5C–E. Noticeably, eukaryotic translation initiation factor 4E binding protein 1 (4EBP1) and EPH receptor A2 (EPHA2), which play important role in progression of cervical cancer [22, 23], particularly in regulating migration and invasion [24, 25], were hyperphosphorylated (Fig. 5B). Especially, 4EBP1 was enriched in cellular senescence pathway (Fig. 5E). Finally, in addition to analyzing the results of omics, we also used the western blot analysis to verify the phosphorylation of key proteins, and the results are shown in Fig. 5F and Supplementary Fig S4A. Compared with oeDDR1 (DOX-) cell, the phosphorylation of 4EBP1 and EPHA2 was remarkable in oeDDR1 (DOX+) cell. DDR1 increased the phosphorylation of key proteins in cervical cancer cell.

### DDR1 influenced downstream phosphorylation signal via GRB2

**Fig. 6:**
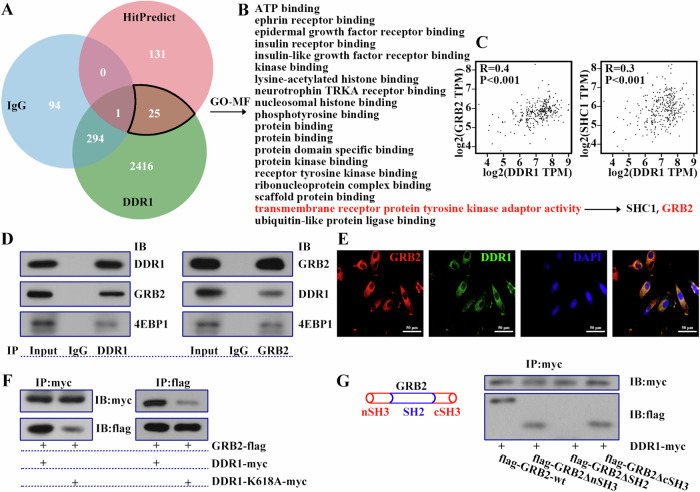
DDR1 influenced downstream phosphorylation signal via GRB2. **A** Veen analysis of the three data sets, including results of co-IP (IgG and DDR1), and the predicted results of HitPredict (http://www.hitpredict.org/). **B** GO-MF analysis results. **C** Correlation of DDR1 and GRB2, DDR1 and SHC1. **D** The co-IP assay was used to verify the combination of DDR1 and GRB2 in SiHa cells. **E** The colocalization of DDR1 and GRB2 in SiHa cells was showed by immunofluorescence double staining. **F** The effect of DDR1 activation on the binding of DDR1 to GRB2 was certificated by co-IP in HEK293T cells. **G** The co-IP assay was used to detect the DDR1 binding site on GRB2 in HEK293T cells. Data were showed as the mean ± standard deviation (*N* = 3). DDR1 discoidin domain receptor 1, GRB2 growth factor receptor bound protein 2, SHC1 SHC Adaptor Protein 1, co-IP co-immunoprecipitation, IgG immunoglobin G, GO-MF Gene Ontology-Molecular Function.

Gupta et al. suggested that DDR1 protein relays signaling via adaptor proteins in colorectal adenocarcinoma and recurrent glioblastoma [26]. We study the adaptor protein of DDR1 in cervical cancer cell. The outcome after co-IP was subjected to mass spectrometry and the results of identification were shown in Fig. 6A. Among the 2736 proteins bound to DDR1, we selected 2441 proteins that specifically bound DDR1. In order to screen more accurately, we introduced the results of HitPredict (http://www.hitpredict.org/), which is a resource of experimentally determined protein-protein interactions. As shown in Fig. 6A, 25 more precise binding proteins were obtained. After GO- Molecular Function analysis, GRB2 and SHC1 were enriched in transmembrane receptor protein tyrosine kinase adaptor activity pathway (Fig. 6B). The correlation of DDR1 and GRB2 was higher than that between DDR1 and SHC1 (Fig. 6C). Therefore, our paper focused on the relationship of DDR1 and GRB2. Following, we carried out co-IP and immunofluorescence assay, and the results demonstrated that DDR1 was combined with GRB2 and co-localized in SiHa cells (Fig. 6D, E). Next, the detailed binding site was detected in HEK293T cells. When DDR1 was deactivated, the combination of DDR1 and GRB2 was significantly weakened (Fig. 6F). It seemed to indicate that the binding of DDR1 to adaptor protein was largely dependent on its activation. Considering the binding region of DDR1 on GRB2, we introduced domain deletion plasmid for co-IP. After SH2 domain deficiency, binding was significantly decreased (Fig. 6G). Together, these results demonstrated that DDR1 bound to GRB2.

### DDR1 accelerated malignant activity and downstream phosphorylation by GRB2

**Fig. 7:**
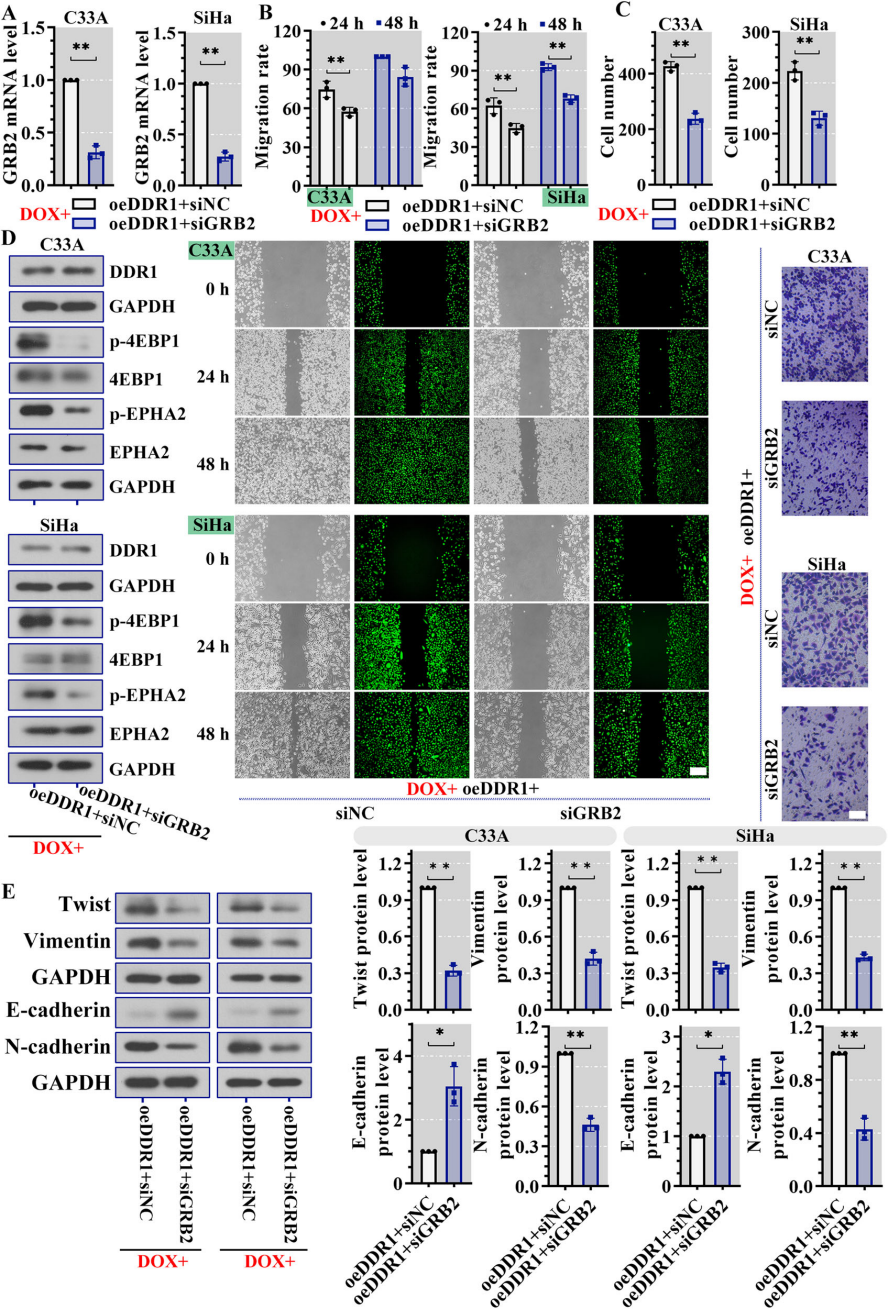
DDR1 accelerated malignant activity and downstream phosphorylation by GRB2. **A** RT-PCR as used to examine the expression of GRB2. **B** Cell migration was detected by wound healing assay (scale bar, 200 μm). **C** The invasion ability of cells was tested by transwell assay (scale bar, 100 μm). **D** Western blot assay was used to measure the phosphorylation of 4EBP1 and EPHA2, and the expression of DDR1. **E** Protein levels of Twist, vimentin, E-cadherin and N-cadherin were measured by western blot assay. Results were displayed as the mean ± standard deviation (*N* = 3). *P < 0.05 was considered statistically significant; **P < 0.01. DDR1 discoidin domain receptor 1, GRB2 growth factor receptor bound protein 2, RT-PCR real-time polymerase chain reaction, 4EBP1 eukaryotic translation initiation factor 4E binding protein 1, EPHA2 EPH receptor A2.

We next asked whether DDR1 played a role through GRB2 in cervical cancer. RT-PCR results showed that GRB2 silencing reduced its mRNA expression in oeDDR1 cells (Fig. 7A). Lessened migration rate and invasive cell number in oeDDR1 cell with siGRB2 transfection suggested that migratory and invasive abilities of cervical cancer cell were decreased (Fig. 7B, C). Compared to control cells, cells with GRB2 silencing showed increased E-cadherin level and reduced N-cadherin, Twist and vimentin level (Fig. 7E). In DDR1 overexpression cell, knockdown GRB2 greatly attenuated the phosphorylation of 4EBP1 and EPHA2, and had no effect on DDR1 expression (Fig. 7D and Supplementary Fig. S4B). Overall, these results demonstrated that DDR1 contributed to the migration, invasion and phosphorylation, that was mediating by GRB2.

## Discussion

Although significant progress has been made in diagnosis and study, cervical cancer is still the main cause of cancer-related death in women. Exploring molecular mechanism of cervical cancer development is a critical challenge.

We studied the role of DDR1 in cervical cancer. The migration, invasion, and EMT of cervical cancer cells were accelerated by DDR1 in vitro and in vivo, evidenced by increased migration rate and invasive cell number, changed EMT indexes, more cell metastasis and number of metastatic nodules, and bigger nodule size. EMT, as a biological process, plays an important role in the progression and metastasis of many solid tumors due to its ability to enhance the migration and invasion of cancer cells [27, 28]. During EMT, cancer cells inappropriately express N-cadherin but lose E-cadherin [29]. The western blot results supported that DDR1 increased the EMT. In line with our conclusion, previous studies proposed that DDR1 enhanced the ability to migrate and invade in various cancers. Han et al. found that DDR1 silencing inhibited the migration and invasion of breast cancer cells [30]. While overexpression of DDR1 caused a significant increase of osteosarcoma cell motility and invasiveness [31].

A pivotal role for SOX2 in the process of cervical cancer has been found in the literatures [21, 32]. However, the exact downstream by which SOX2 modulated were largely unknown. Considering previous study and website predicted results, we found that SOX2 has the ability to regulate transcription and combines with promoter of DDR1. While DDR1 was increased in cervical cancer that was the outcome after analyzing data from GEO and GEPIA. Thus, the function of DDR1 in cervical cancer was further assessed in this study. Firstly, increased DDR1 and SOX2 were verified in cervical cancer samples. We observed that SOX2 and DDR1 were positively correlated and SOX2 promoted transcription of DDR1. It seemed to suggest that DDR1 was the downstream of SOX2, when SOX2 acted as an oncogene in cervical cancer.

With the current results, we demonstrated that DDR1 was transcriptionally regulated by SOX2 and facilitated the transfer of cervical cancer cell. But the lack of knowledge on downstream of DDR1 is a problem to be solved.

DDR1, as a kind of receptor tyrosine kinase, consists of an extracellular ligand binding domain, a single transmembrane domain, and an intracellular kinase domain [33]. When DDR1 is activated by receptor-specific ligands, it recruits and activates multifarious downstream signaling proteins [15]. Therefore, the phosphoproteomics of cervical cancer cell after oeDDR1 overexpression was focused on. DDR1 modulated the phosphorylation of many downstream targets, including 4EBP1 and EPHA2. 4EBP1 and EPHA2 play important roles in cervical cancer progression [22, 23]. In apoptotic human cervical cancer HeLa cells, Rho et al. found the diminished phosphorylation of 4EBP1 [34]. Significant expression of p-4EBP1 was related to high-grade tumors and poor prognosis of patients with ovarian carcinoma [35]. In addition, overexpression of 4EBP1 promoted the migration and invasion and enhanced phosphorylation of 4EBP1 aggravated the proliferation and metastasis of cancer [24, 36]. Li et al, also proposed that 4EBP1 increased the expression of N-cadherin, matrix metalloproteinase-2 and 9, and reduced E-cadherin [36]. EPHA2 belongs to the Eph receptors family, and its phosphorylation causes the decreased cell-extracellular matrix attachment [37], while inhibition of p-EPHA2 is accompanied by reduced vimentin and increased E-cadherin [38]. Knockdown of EPHA2 inhibited the migration and invasion of cancer cells [25, 39]. In summary, 4EBP1 and EPHA2 contribute to migration and invasion, as well as facilitating the production of matrix metalloproteinase. However, the exactly mechanisms need to be further explored. Notably, the phosphorylation of 4EBP1 and EPHA2 in oeDDR1 cervical cancer cells was authenticated and verified by phosphoproteomics and western blot assay. These results indicated that DDR1 changed the downstream phosphorylation signals, thereby regulating the development of cervical cancer.

Adapter proteins containing SH2 domains bind receptor tyrosine kinases to downstream signal transducing molecules [40]. Thus, our next work was devoted to finding the adapter protein that linked DDR1 and downstream signals in cervical cancer. The SH2 domain of GRB2 and the fact that GRB2, which was identified as a protein that bound to DDR1, was enriched in many signal pathways make it stand out. Our results not only suggested that DDR1 combined with GRB2 assuredly, but also revealed that to some extent, the binding of DDR1 to GRB2 was depended on the activation of DDR1. In co-IP assay, the band loss in cells co-transfected with DDR1 and GRB2ΔSH2 demonstrated that SH2 domain of GRB2 was responsible for interaction with DDR1. Previous evidence also found that SH2 domain existed in adapter proteins, such as NCK adaptor protein 2 and protein tyrosine phosphatase non-receptor type 11, was responsible for binding with DDR1 and crucial for downstream signal [41, 42]. In addition, the reduced migration rate and invasive cell number, and attenuated phosphorylation of 4EBP1 and EPHA2 supported that DDR1 influenced the migration and invasion of cervical cancer via GRB2.

In summary, the present study suggests that DDR1 bound to the SH2 domain of GRB2, thereby affecting downstream phosphorylation signals and ultimately exacerbating the migration, invasion and EMT of cervical cancer cells. Furthermore, DDR1 was transcriptionally regulated by SOX2 and might be a probable biomarker in cervical cancers.

## Supplementary information

Supplementary information for materials and methods

Supplementary file 1. Cervical cancer cells were transfected successfully

Supplementary file 2. Cervical cancer cells were successfully infected with lentivirus (inducible expression system)

Supplementary file 3. DDR1 promoted the metastasis of cervical cancer cell

Supplementary file 4. Effect of DDR1 on downstream phosphorylation signal

Supplementary file figure legends

Supplementary files-western blots

## Data Availability

The datasets used and/or analyzed during the current study are available from the corresponding author on reasonable request.
