# Supplementary material for: DDR1 promotes metastasis of cervical cancer and downstream phosphorylation signal via binding GRB2
Source: Cell Death Dis. 2024 Nov 20;15(11):849. doi: 10.1038/s41419-024-07212-5 (PMC11579010; doi:10.1038/s41419-024-07212-5)
Supplement: Supplementary file 1 — Supplementary information for materials and methods [file 41419_2024_7212_MOESM1_ESM.docx]

## Cell culture

Cervical cancer cells were cultured in Dulbecco’s modified Eagle’s medium (Wuhan Servicebio Technology Co., Ltd., Hubei, China) plus 10% fetal bovine serum (FBS, Zhejiang Tianhang Biotechnology Co., Ltd., Huzhou, China). The medium used for 293T cell culture was composed of Minimum Essential Medium (Solarbio, Beijing, China) supplemented with 10% FBS. All cells were maintained in a 5% CO_2_ incubator at 37°C.

## Animal feeding environment

At room temperature of 22 ± 1 °C and humidity of 45 - 55%, female BALB/c nude mice (six-week-old) maintained in a light-dark cycle (12 h light and 12 h darkness) and were given free access to diet and water.

## Details for DNA pull-down

Briefly, biotin-labelled target DNA probe was mixed with magnetic beads for 30 minutes. Nucleoprotein was prepared and the protein concentration was quantified. Protein was incubated with DNA-coupled magnetic beads at 4 °C for 1 h, and subsequently the compound was placed on a magnetic rack to collect magnetic beads. After elution in protein elution buffer for 120 min at 37 °C, protein was analyzed by western blot assay.

## Antibody information

| Name | Vendor | Cat. No. | Experiment |
| --- | --- | --- | --- |
| DDR1 | Proteintech Group, Inc. | 10536-1-AP | Immunohistochemistry |
| goat anti-rabbit IgG (H+L) secondary antibody | Thermo Scientific | 31460 | Immunohistochemistry |
| DDR1 | Cell Signaling Technology | 5583 | Immunofluorescent staining |
| GRB2 | Santa Cruz Biotechnology, Inc. | sc-8034 | Immunofluorescent staining |
| Goat Anti-Rabbit IgG H&L (FITC) | Abcam | ab6717 | Immunofluorescent staining |
| Goat Anti-Mouse IgG H&L (Cy3 ®) preadsorbed | Abcam | ab97035 | Immunofluorescent staining |
| DDR1 | Cell Signaling Technology | 5583 | Western blot |
| SOX2 | Cell Signaling Technology | 2748 | Western blot |
| p-4EBP1 | Affinity Biosciences | AF3830 | Western blot |
| 4EBP1 | Abcam | ab32024 | Western blot |
| p-EPHA2 | Affinity Biosciences | AF7279 | Western blot |
| EPHA2 | Affinity Biosciences | AF5238 | Western blot |
| E-cadherin | Affinity Biosciences | AF0131 | Western blot |
| N-cadherin | ABclonal | A19083 | Western blot |
| vimentin | ABclonal | A19607 | Western blot |
| Twist | Proteintech Group, Inc. | 25465-1-AP | Western blot |
| GAPDH | Proteintech Group, Inc. | 60004-1-Ig | Western blot |
| GRB2 | ABclonal | A19059 | co-IP |
| flag | ABclonal | AE063 | co-IP |
| myc | ABclonal | AE010 | co-IP |
| Goat Anti-Rabbit IgG/HRP | Solarbio | SE134 | co-IP&Western blot |
| Goat Anti-Mouse IgG/HRP | Solarbio | SE131 | co-IP&Western blot |

## Primer sequences

GRB2 forward primer: 5'-TACTTCCTCTGGGTGGTG-3'

GRB2 reverse primer: 5'-GTATGTCGGCTGCTGTG-3'

SOX2 forward primer: 5'-ATGCACCGCTACGACGTGAG-3'

SOX2 reverse primer: 5'-GCCCTGGAGTGGGAGGAAGA-3'

DDR1 forward primer: 5'-CCGACTGGTTCGCTTCTAC-3'

DDR1 reverse primer: 5'-GTCCGCCCACGGTATGT-3'
