# Supplementary figures and images for: DDR1 promotes metastasis of cervical cancer and downstream phosphorylation signal via binding GRB2
Source: Cell Death Dis. 2024 Nov 20;15(11):849. doi: 10.1038/s41419-024-07212-5 (PMC11579010; doi:10.1038/s41419-024-07212-5)

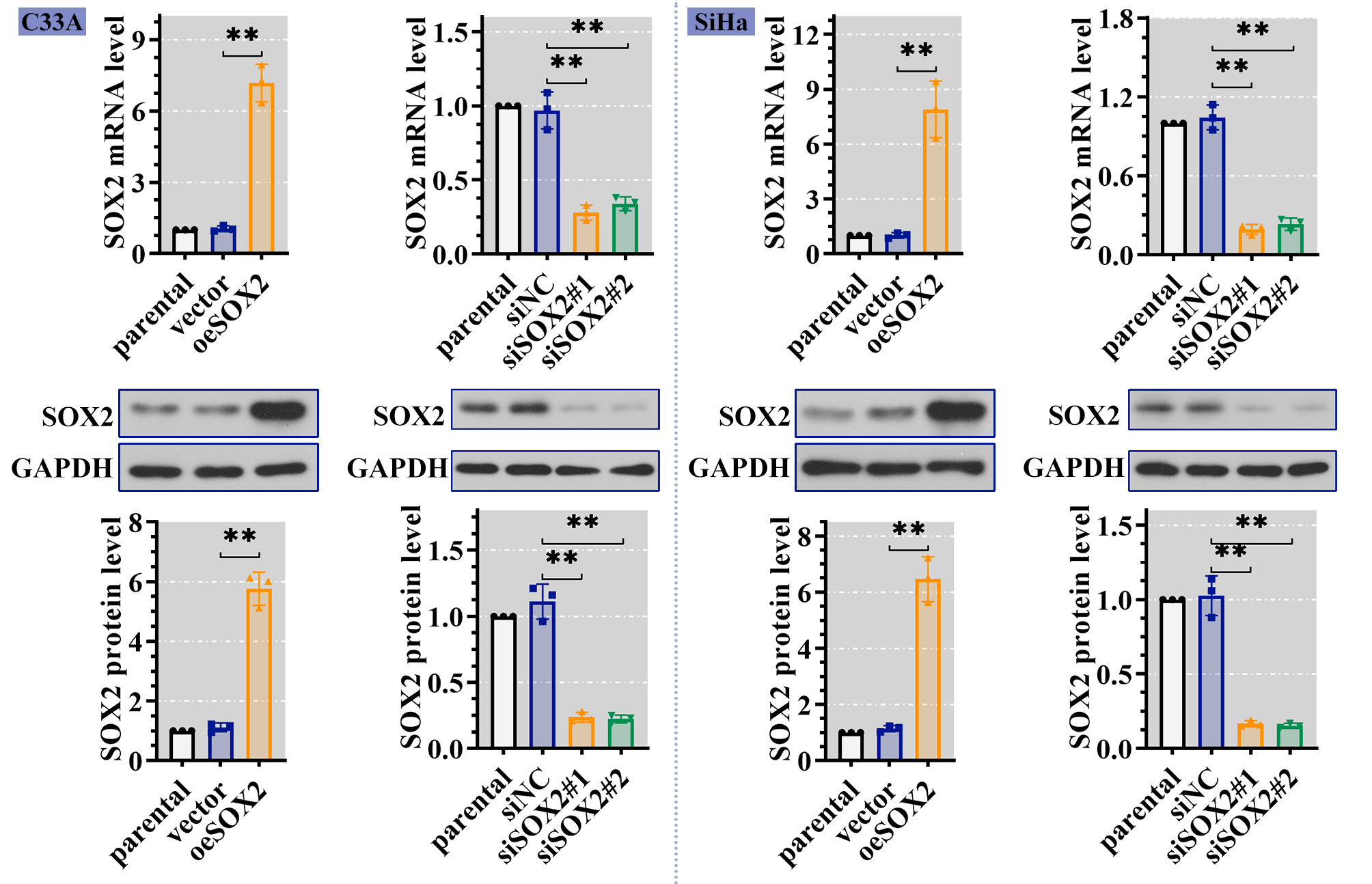

Supplement: Supplementary file 2 — Supplementary file 1. Cervical cancer cells were transfected successfully [file 41419_2024_7212_MOESM2_ESM.tif]

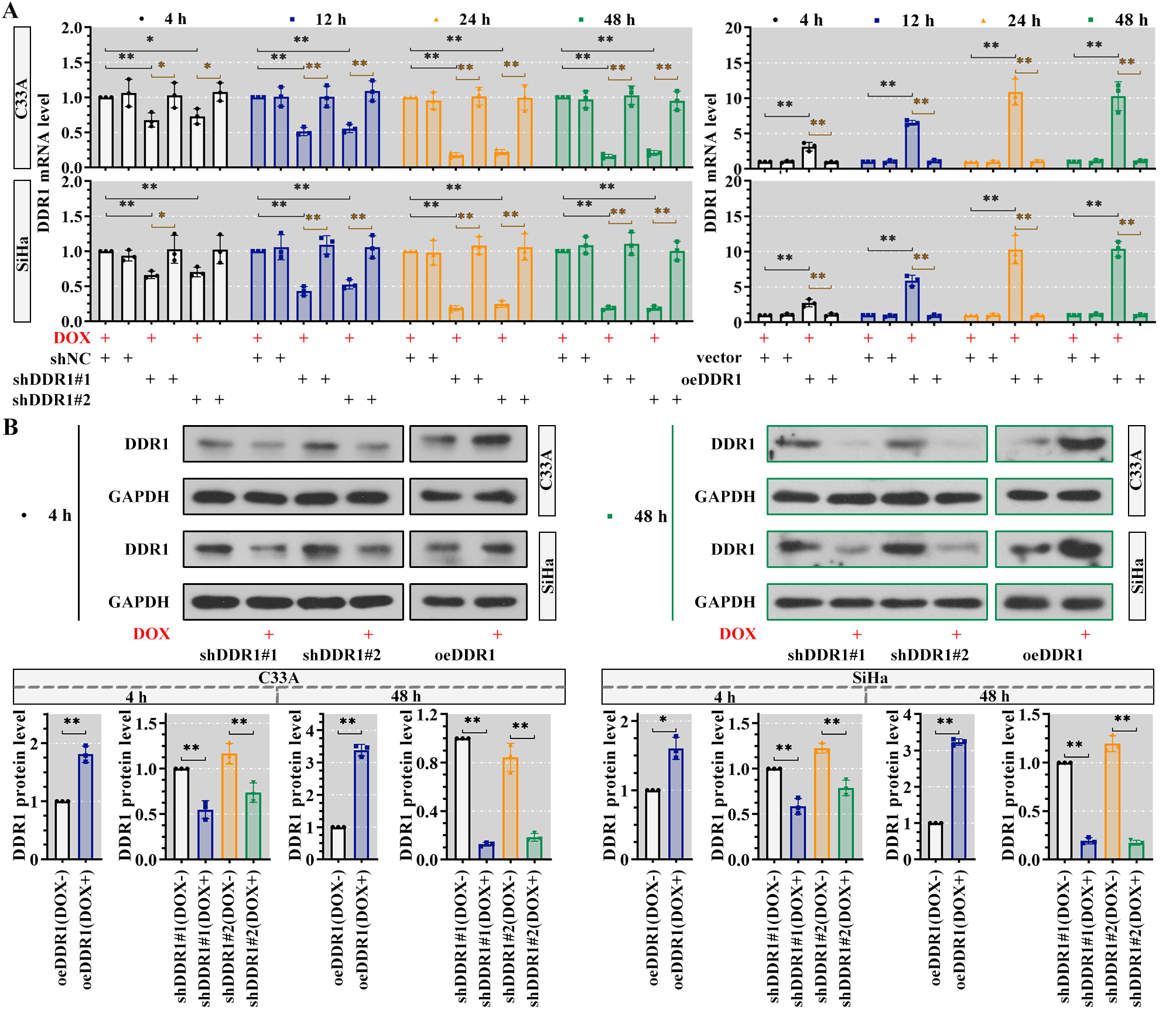

Supplement: Supplementary file 3 — Supplementary file 2. Cervical cancer cells were successfully infected with lentivirus (inducible expression system) [file 41419_2024_7212_MOESM3_ESM.tif]

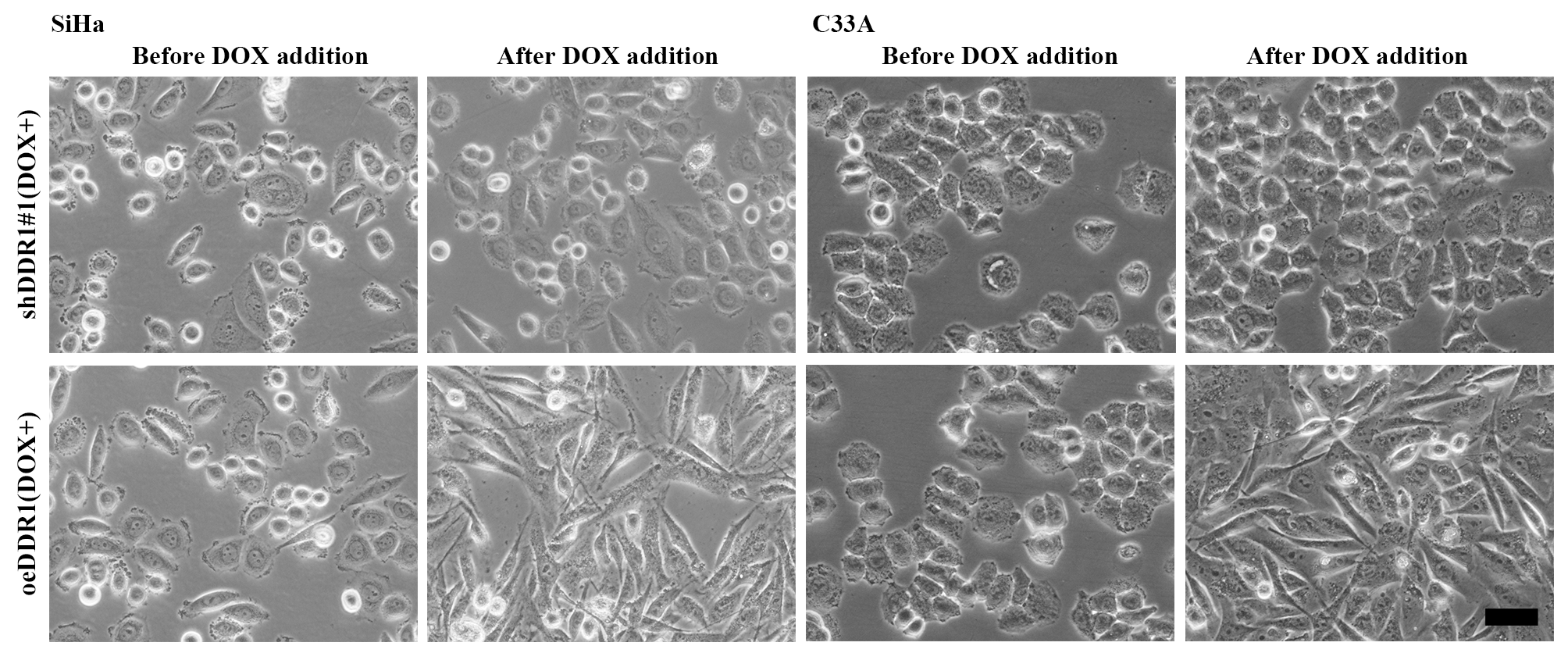

Supplement: Supplementary file 4 — Supplementary file 3. DDR1 promoted the metastasis of cervical cancer cell [file 41419_2024_7212_MOESM4_ESM.tif]

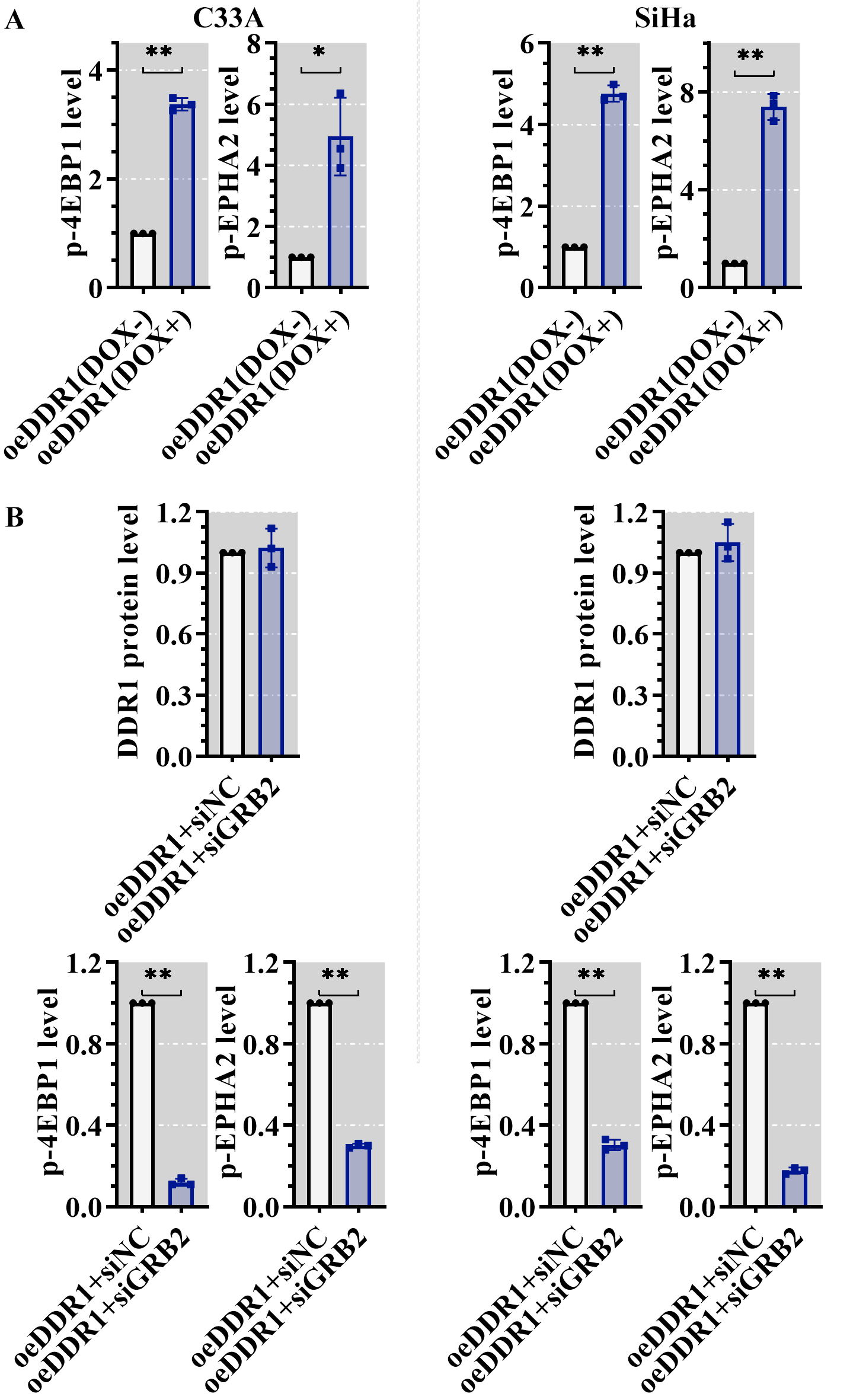

Supplement: Supplementary file 5 — Supplementary file 4. Effect of DDR1 on downstream phosphorylation signal [file 41419_2024_7212_MOESM5_ESM.tif]
