# Supplementary material for: DDR1 promotes metastasis of cervical cancer and downstream phosphorylation signal via binding GRB2
Source: Cell Death Dis. 2024 Nov 20;15(11):849. doi: 10.1038/s41419-024-07212-5 (PMC11579010; doi:10.1038/s41419-024-07212-5)
Supplement: Supplementary file 6 — Supplementary file figure legends [file 41419_2024_7212_MOESM6_ESM.docx]

# Supplementary file figure legends

## Supplementary file 1. Cervical cancer cells were transfected successfully

The expression of SOX2 was measured by RT-PCR and western blot assay.

Results were presented as the mean ± standard deviation (N = 3). *P < 0.05 was considered statistically significant; ** P < 0.01.

SOX2, SRY-Box transcription factor 2; RT-PCR, real-time polymerase chain reaction.

## Supplementary file 2. Cervical cancer cells were successfully infected with lentivirus (inducible expression system)

A&B. RT-PCR and western blot was used to examine the expression of DDR1 at different time points.

Results were showed as the mean ± standard deviation (N = 3). *P < 0.05 was considered statistically significant; ** P < 0.01.

DDR1, discoidin domain receptor 1; RT-PCR, real-time polymerase chain reaction.

## Supplementary file 3. DDR1 promoted the metastasis of cervical cancer cell

The change of cellular morphology was shown (scale bar, 50 μm).

DDR1, discoidin domain receptor 1.

## Supplementary file 4. Effect of DDR1 on downstream phosphorylation signal

A& B. Western blot was used to detect the level of p-4EBP1, 4EBP1, p-EPHA2, EPHA2 and DDR1.

Results were showed as the mean ± standard deviation (N = 3). *P < 0.05 was considered statistically significant; ** P < 0.01.

DDR1, discoidin domain receptor 1; p-4EBP1, phosphorylation-eukaryotic translation initiation factor 4E binding protein 1; p-EPHA2, phosphorylation-EPH receptor A2.
