# Supplementary material for: DDR1 promotes metastasis of cervical cancer and downstream phosphorylation signal via binding GRB2
Source: Cell Death Dis. 2024 Nov 20;15(11):849. doi: 10.1038/s41419-024-07212-5 (PMC11579010; doi:10.1038/s41419-024-07212-5)

Fig. 1.

Fig. 1C

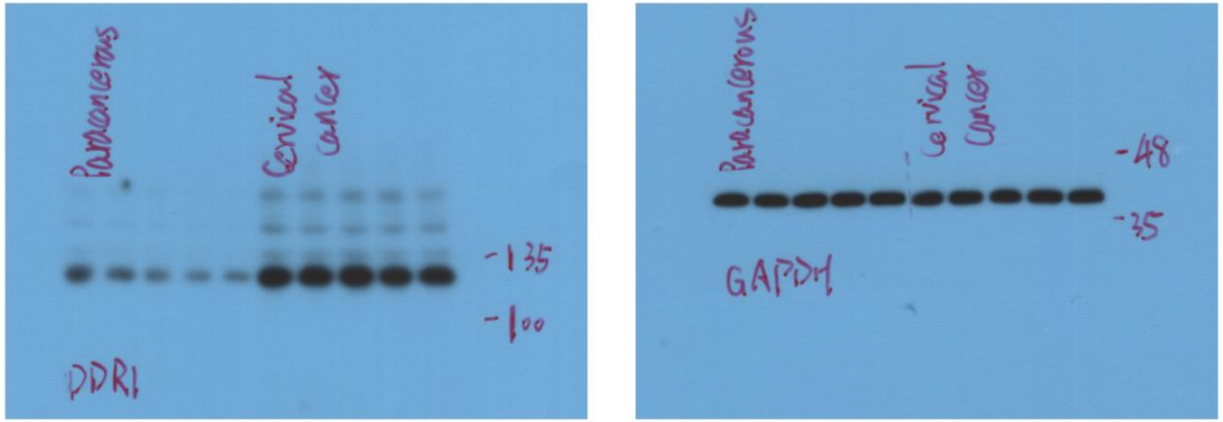

Paracancerous
Cervical cancer
-135
-100
DDR1
Paracancerous
Cervical cancer
-48
-35
GAPDH

Fig. 2.

Fig. 2E

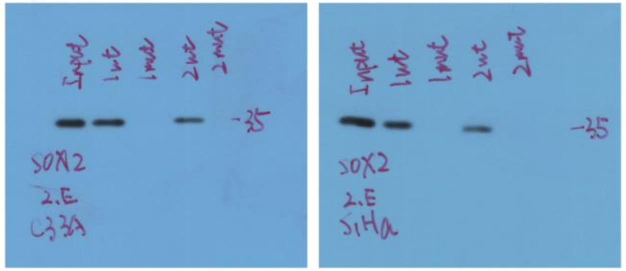

Fig. 2F

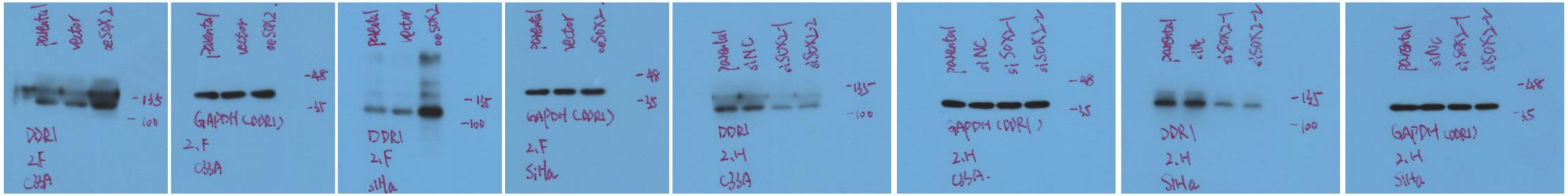

Fig. 3.

Fig. 3C

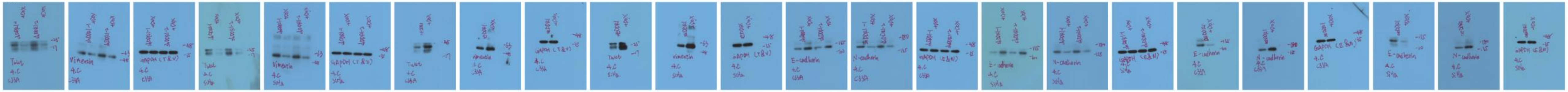

Fig. 5.

Fig. 5F

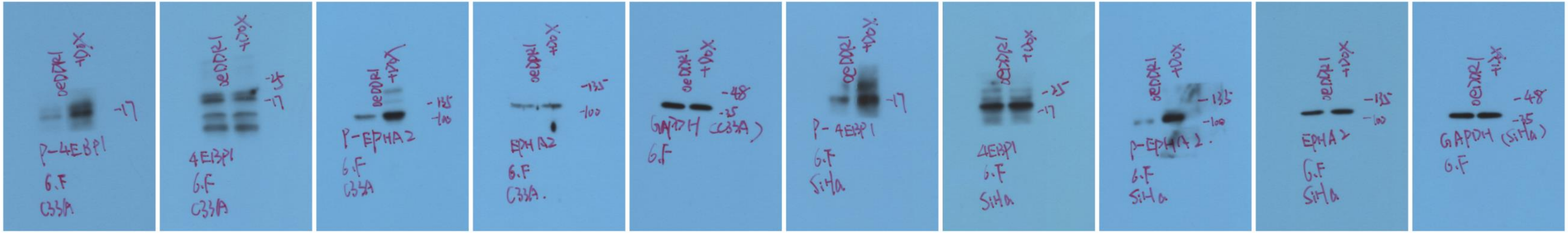

Fig. 6.

Fig. 6D

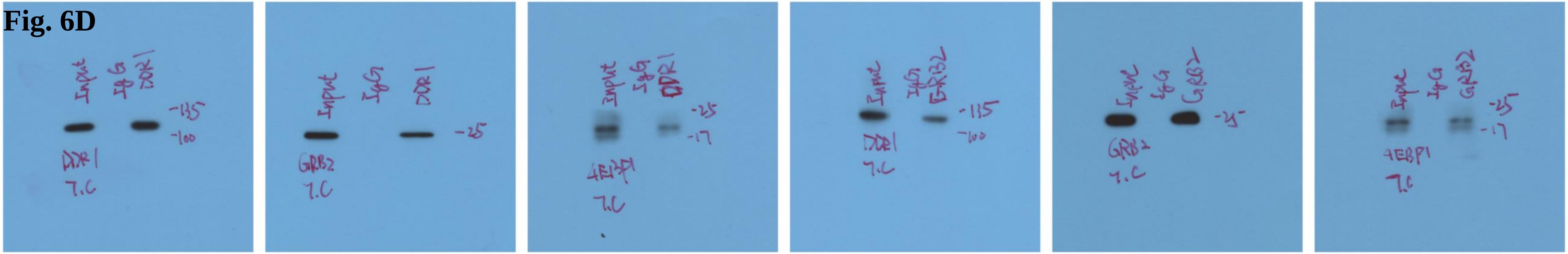

Fig. 6F

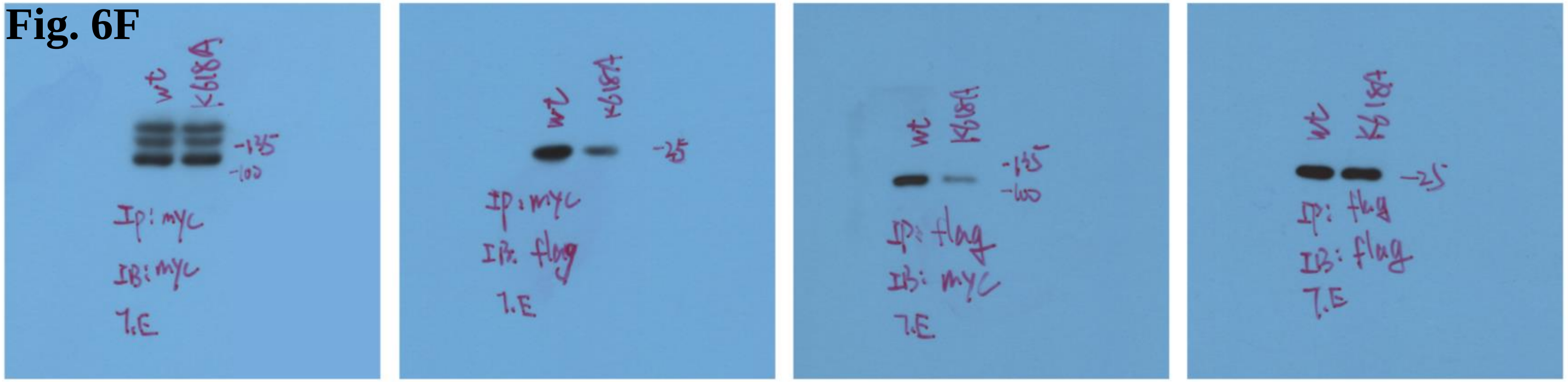

Fig. 6G

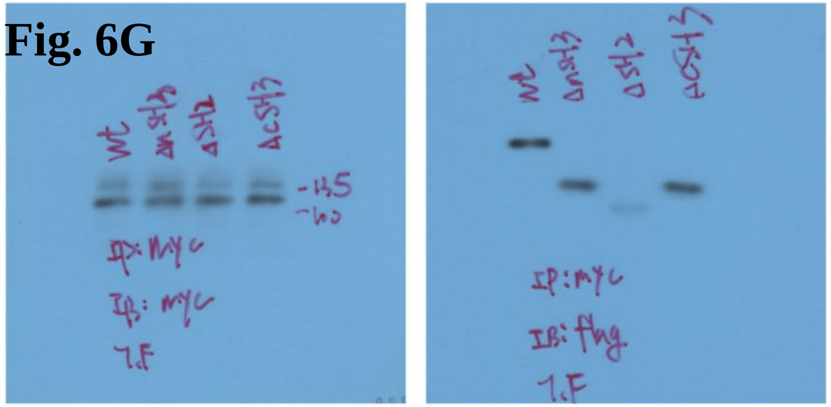

Fig. 7.

Fig. 7D

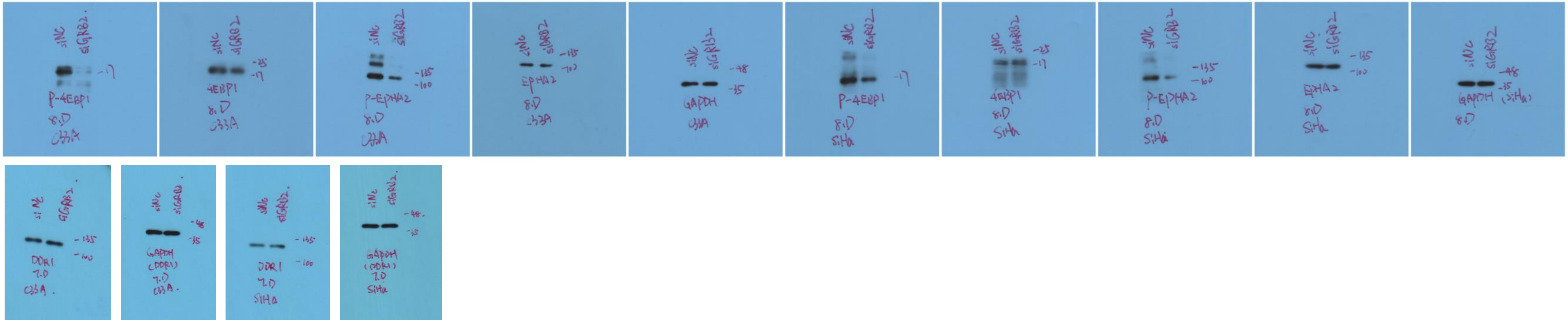

Fig. 7E

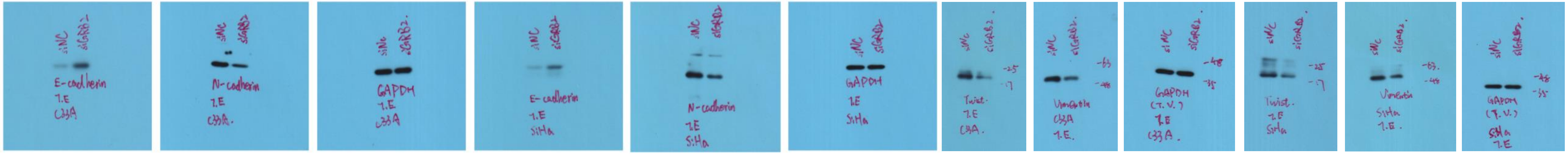

Supplementary file 1

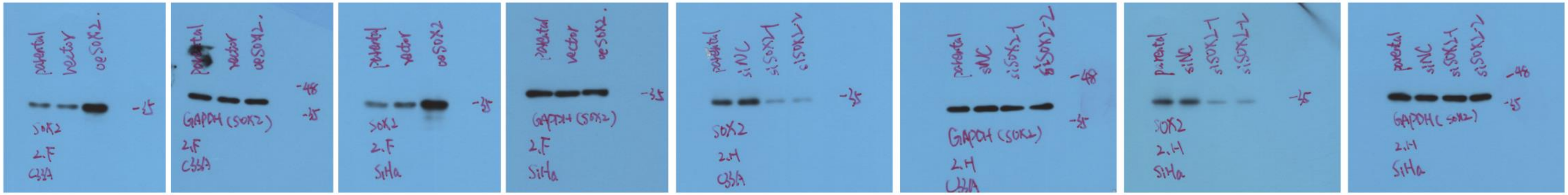

Supplementary file 2

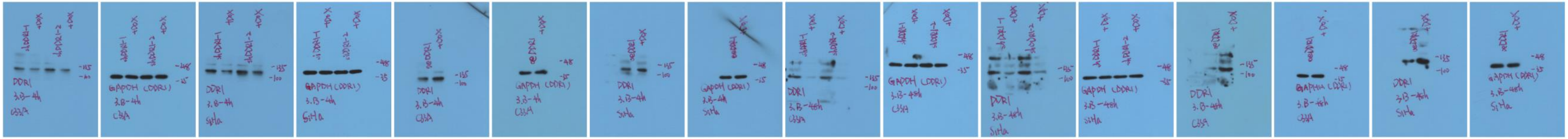

Supplement: Supplementary file 7 — Supplementary files-western blots [file 41419_2024_7212_MOESM7_ESM.pdf]
